# Revisiting Anti-tuberculosis Therapeutic Strategies That Target the Peptidoglycan Structure and Synthesis

**DOI:** 10.3389/fmicb.2019.00190

**Published:** 2019-02-11

**Authors:** Maria João Catalão, Sérgio R. Filipe, Madalena Pimentel

**Affiliations:** ^1^ Research Institute for Medicines (iMed.ULisboa), Faculty of Pharmacy, Universidade de Lisboa, Lisbon, Portugal; ^2^ UCIBIO-REQUIMTE, Departamento de Ciências da Vida, Faculdade de Ciências e Tecnologia, Caparica, Portugal; ^3^ Laboratory of Bacterial Cell Surfaces and Pathogenesis, Instituto de Tecnologia Química e Biológica António Xavier, Universidade Nova de Lisboa, Oeiras, Portugal

**Keywords:** mycobacteria, cell wall, tuberculosis, antibiotic resistance, peptidoglycan, β-lactams, mycobacteriophage lysis enzymes

## Abstract

Tuberculosis (TB), which is caused by *Mycobacterium tuberculosis* (*Mtb*), is one of the leading cause of death by an infectious diseases. The biosynthesis of the mycobacterial cell wall (CW) is an area of increasing research significance, as numerous antibiotics used to treat TB target biosynthesis pathways of essential CW components. The main feature of the mycobacterial cell envelope is an intricate structure, the mycolyl-arabinogalactan-peptidoglycan (mAGP) complex responsible for its innate resistance to many commonly used antibiotics and involved in virulence. A hallmark of mAGP is its unusual peptidoglycan (PG) layer, which has subtleties that play a key role in virulence by enabling pathogenic species to survive inside the host and resist antibiotic pressure. This dynamic and essential structure is not a target of currently used therapeutics as *Mtb* is considered naturally resistant to most β-lactam antibiotics due to a highly active β-lactamase (BlaC) that efficiently hydrolyses many β-lactam drugs to render them ineffective. The emergence of multidrug- and extensive drug-resistant strains to the available antibiotics has become a serious health threat, places an immense burden on health care systems, and poses particular therapeutic challenges. Therefore, it is crucial to explore additional *Mtb* vulnerabilities that can be used to combat TB. Remodeling PG enzymes that catalyze biosynthesis and recycling of the PG are essential to the viability of *Mtb* and are therefore attractive targets for novel antibiotics research. This article reviews PG as an alternative antibiotic target for TB treatment, how *Mtb* has developed resistance to currently available antibiotics directed to PG biosynthesis, and the potential of targeting this essential structure to tackle TB by attacking alternative enzymatic activities involved in *Mtb* PG modifications and metabolism.

## Introduction

According to the latest report available from the World Health Organization (WHO), it is estimated that in 2017, there were about 10.3 million new cases of TB worldwide and about 1.8 million people died from this infection. The emergence of multidrug-resistant (MDR) and extensive drug-resistant (XDR) strains to the available antibiotics is a worldwide public health problem of increasing importance, with a treatment success rate of only about 50%, which decreases to 23% in the case of XDR-TB (World Health Organization, 2010, 2011, 2014, 2017; Horsburgh et al., 2015). The lack of effective treatment regimens against MDR-TB and XDR-TB isolates has highlighted the potential of repurposing existing antibiotic options in alternative and innovative ways (Mainardi et al., 2011) as all drugs, except for bedaquiline and delamanid, which are currently used to treat TB, were approved several years ago, demonstrating the complexity of TB drug development (Wong et al., 2013; Keener, 2014; Diacon et al., 2016).

A hallmark of *Mtb*, the causative agent of TB, as a successful pathogen is its intricate CW (Brennan and Nikaido, 1995; Jankute et al., 2015) that has been associated with the genetic differences among human lineages of *Mtb* (Portevin et al., 2011). The core of the mycobacteria cell envelope is composed of three main structures: (1) the characteristic long-chain mycolic acids (MA); (2) a highly branched arabinogalactan (AG) polysaccharide; and (3) a very cross-linked and modified meshwork of PG. The entire complex, referred to as mycolyl-arabinogalactan-peptidoglycan (mAGP) (Brennan and Nikaido, 1995; Alderwick et al., 2015; Jankute et al., 2015), is essential for *Mtb* viability, virulence, and persistence and can modulate the innate immune response (Brennan and Nikaido, 1995; Stanley and Cox, 2013; Jankute et al., 2015). In addition, it acts as an impregnable external barrier responsible for the intrinsic resistance of *Mtb* to several drugs (Nikaido, 1994; Gygli et al., 2017; Nasiri et al., 2017). The essential nature of CW synthesis and assembly has rendered the mycobacterial CW as the most extensively exploited target of anti-TB drugs (Wong et al., 2013; Bhat et al., 2017). Ethambutol, isoniazid, and ethionamide successfully target the synthesis of the various components of mAGP (Jackson et al., 2013), and resistance to these drugs, which is mediated by the accumulation of chromosomal mutations in genes involved in CW biosynthesis pathways, can arise under selective pressure of antibiotic use (Eldholm and Balloux, 2016; Gygli et al., 2017; Nasiri et al., 2017). *Mtb* has been considered innately resistant to most β-lactam antibiotics that target PG biosynthesis due to (1) a highly active β-lactamase (BlaC) that efficiently inactivates many β-lactams (Wang et al., 2006; Hugonnet and Blanchard, 2007) and (2) the fact that a large proportion of the CW PG is cross-linked by non-classical l,d-transpeptidases, which are intrinsically impervious to these antibiotics (Lavollay et al., 2008; Cordillot et al., 2013). Widespread antibiotic resistance in *Mtb*, in combination with the lack of progress in developing new effective treatments, is threatening the ability of tackling the outcomes caused by highly resistant *Mtb* strains. This highlights the need of considering alternative therapeutic schemes to combat the global increase in resistance to the current anti-TB regimens. This review summarizes the current knowledge about the mechanisms employed by mycobacteria to circumvent the activity of currently available antibiotics that target PG biosynthesis with an emphasis on recent advancements regarding the efficacy of carbapenems, a more recent class of extended-spectrum β-lactams against highly drug-resistant *Mtb* clinical strains, and the potential application of mycobacteriophage-encoded lysis proteins to kill mycobacteria by weakening the CW.

## Impact of the Atypical Mycobacterial PG Structure on Resistance to Antibiotics that Target PG Biosynthesis

A distinctive feature of the mycobacterial CW is its unusual PG layer (Alderwick et al., 2015; Jankute et al., 2015), which is essential for survival of *Mtb* and that is linked with the exceptional immunogenic activity associated with the CW. The PG macromolecule contains a number of unique subtleties that enable *Mtb* to survive inside the host and resist different antibiotics (Gygli et al., 2017; Nasiri et al., 2017). The PG layer of *Mtb* is surrounded by other layers dominated by lipids, carbohydrates, and phosphatidyl-myo-inositol-based lipoglycans that provide a permeability barrier against hydrophilic drugs (Nikaido, 1994; Brennan and Nikaido, 1995; Hoffmann et al., 2008). PG acts as a pro-inflammatory inducer that is hypothetically masked within the mAGP complex (Brennan and Nikaido, 1995; Jankute et al., 2015), which constitutes the major structural component of the cell envelope. Access of antibiotics that target PG biosynthesis is critical for their efficacy, and it is now assumed that several pathogenic bacteria have developed different strategies to hide PG (Atilano et al., 2011, 2014), thus circumventing their antibacterial activity. Mycobacterial PG forms the basal layer of the mAGP complex, where glycan chains composed of alternating *N*-acetylglucosamine (Glc*N*Ac) and modified *N*-acetylmuramic acid (Mur*N*Ac) residues, linked in a β (1 → 4) configuration (Alderwick et al., 2015), are interconnected through oligopeptides. The muramic acid residues in *Mtb* are found containing a combination of *N*-acetyl and *N*-glycolyl derivatizations. In the latter case, the *N*-acetyl group present in Mur*N*Ac residues has been oxidized to an *N*-glycolyl group through the action of the enzyme *N*-acetyl muramic acid hydroxylase (NamH) to form Mur*N*Gly (Raymond et al., 2005). Although the precise function of the *N*-glycolyl modification, a structural modification that is unique to mycobacteria (and closely related genera) is yet to be elucidated, it has been hypothesized that it contributes to: (1) the stability of the mycobacterial CW, by strengthening the mesh-like structure of the PG layer providing sites for additional hydrogen bonding between different parts of the PG macromolecule (Brennan and Nikaido, 1995); (2) the increase of β-lactam resistance (Raymond et al., 2005); (3) the protection of bacteria from degradation *via* lysozyme (Raymond et al., 2005); and (4) the overall innate immune response triggered by the CW of mycobacteria, as the glycolylated form of the muramyl dipeptide is an important contributor to the unusual immunogenicity of mycobacteria. This component of the mycobacterial PG is a strong inducer of NOD2-mediated host responses (Coulombe et al., 2009; Schenk et al., 2016), although playing a limited role in the pathogenesis of *Mtb* infection (Hansen et al., 2014). Beside the contribution of glycolylated muramic acid residues to the overall host-mycobacteria interaction, *Mtb* PG-derived muropeptides released by the action of a group of enzymes called “resuscitation-promoting factors,” encoded by the *rpf* genes have also been associated with β-lactam and vancomycin tolerance and increased outer membrane (OM) impermeability (Kana et al., 2010; Wivagg and Hung, 2012). The pentapeptide chains of the mycobacterial PG can also be modified by amidation, glycylation, or methylation (Mahapatra et al., 2005), which contributes to its resistance to endopeptidase activity of PG hydrolases (Lavollay et al., 2008). However, the functional significance of these modifications for *Mtb* drug resistance is unknown.

**Figure 1:**
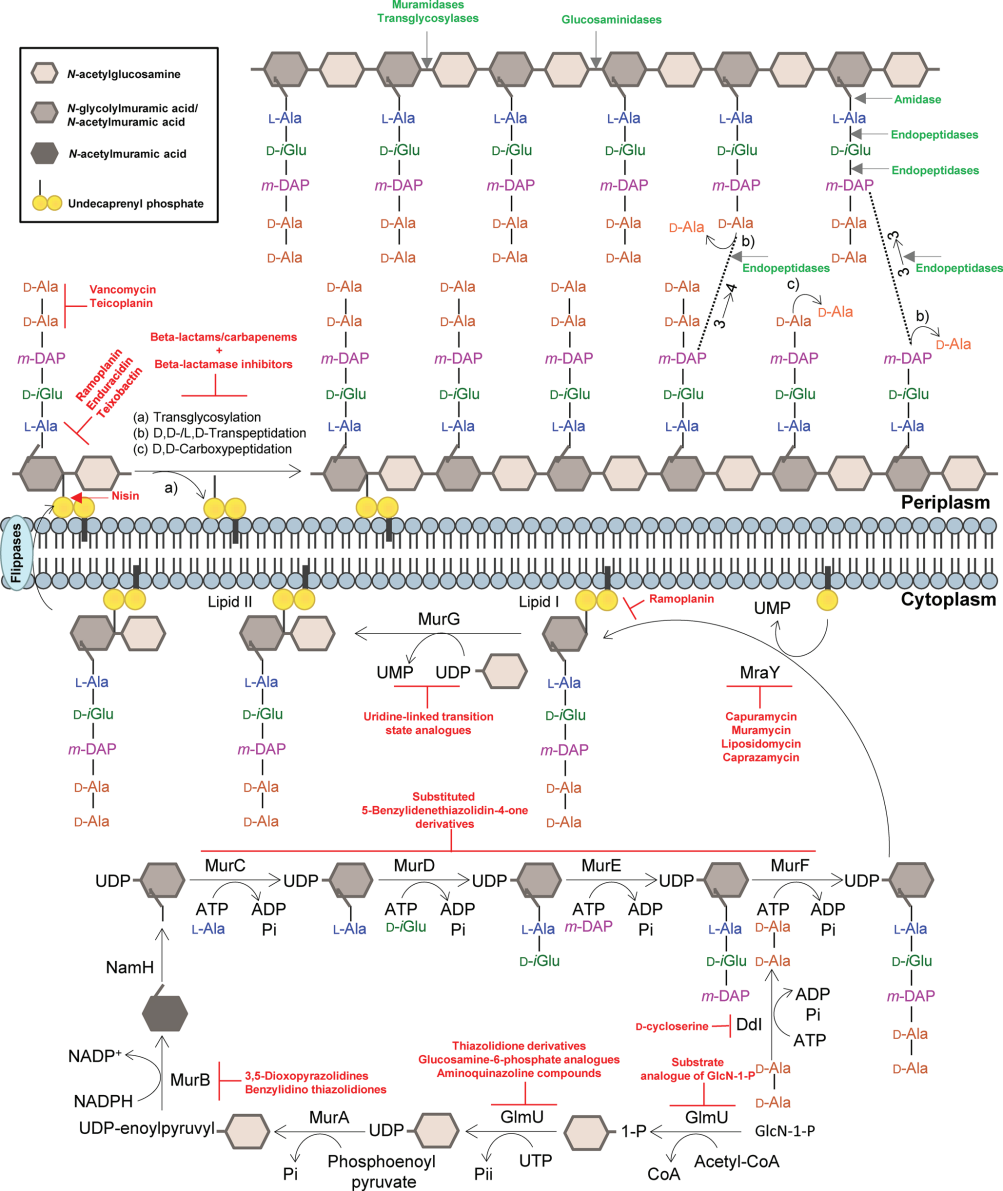
Summary of the mycobacterial peptidoglycan biosynthesis pathway. The peptidoglycan precursors are produced in the cytoplasm, and peptidoglycan monomeric units are assembled in the inner leaflet of the cytoplasmic membrane. Polymerization and cross-linking of tetrapeptide side chains take place at the periplasm. Inhibitors of the peptidoglycan biosynthetic enzymes are colored in red, and peptidoglycan bonds that are targeted by mycobacteriophage endolysins are colored in green. Adapted from Abrahams and Besra, 2018, with permission.

The mature PG architecture is also marked by a high degree of direct peptide cross-links, a characteristic that is not frequently found in other bacteria. Overall, 80% of the peptides are cross-linked in two types of linkages in order to maintain the complexity of the mycobacterial cell envelope during growth and under non-replicating conditions (Lavollay et al., 2008). Mycobacterial PG cross-linking is catalyzed by d,d-transpeptidases (penicillin-binding proteins) and typically by the combined action of non-classical l,d-transpeptidases (Ldts) and d,d-carboxypeptidases. The action of these enzymes results in PG peptides, which connect neighboring glycan chains, that are linked through 4 → 3 (d-Ala-mDAP) and 3 → 3 (mDAP-mDAP) linkages, respectively (Figure 1; Lavollay et al., 2008). The latter set of proteins contributes to the intrinsic resistance to β-lactams and provides protection from PG endopeptidases (Lavollay et al., 2008; Cordillot et al., 2013). Another unique feature of mycobacterial PG is that it provides the attachment site for AG (which is catalyzed by the Lcp1 phosphotransferase) (McNeil et al., 1990; Baumgart et al., 2016; Grzegorzewicz et al., 2016; Harrison et al., 2016), a highly branched molecule assembled from arabinofuranose and galactofuranose monosaccharides, which overlays the PG and that can also preclude PG synthesis from being targeted by β-lactams (Schubert et al., 2017).

## Mycobacterial Intrinsic Resistance to Antibiotics that Target PG Biosynthesis: A New Trick for an Old Dogma

**Figure 2:**
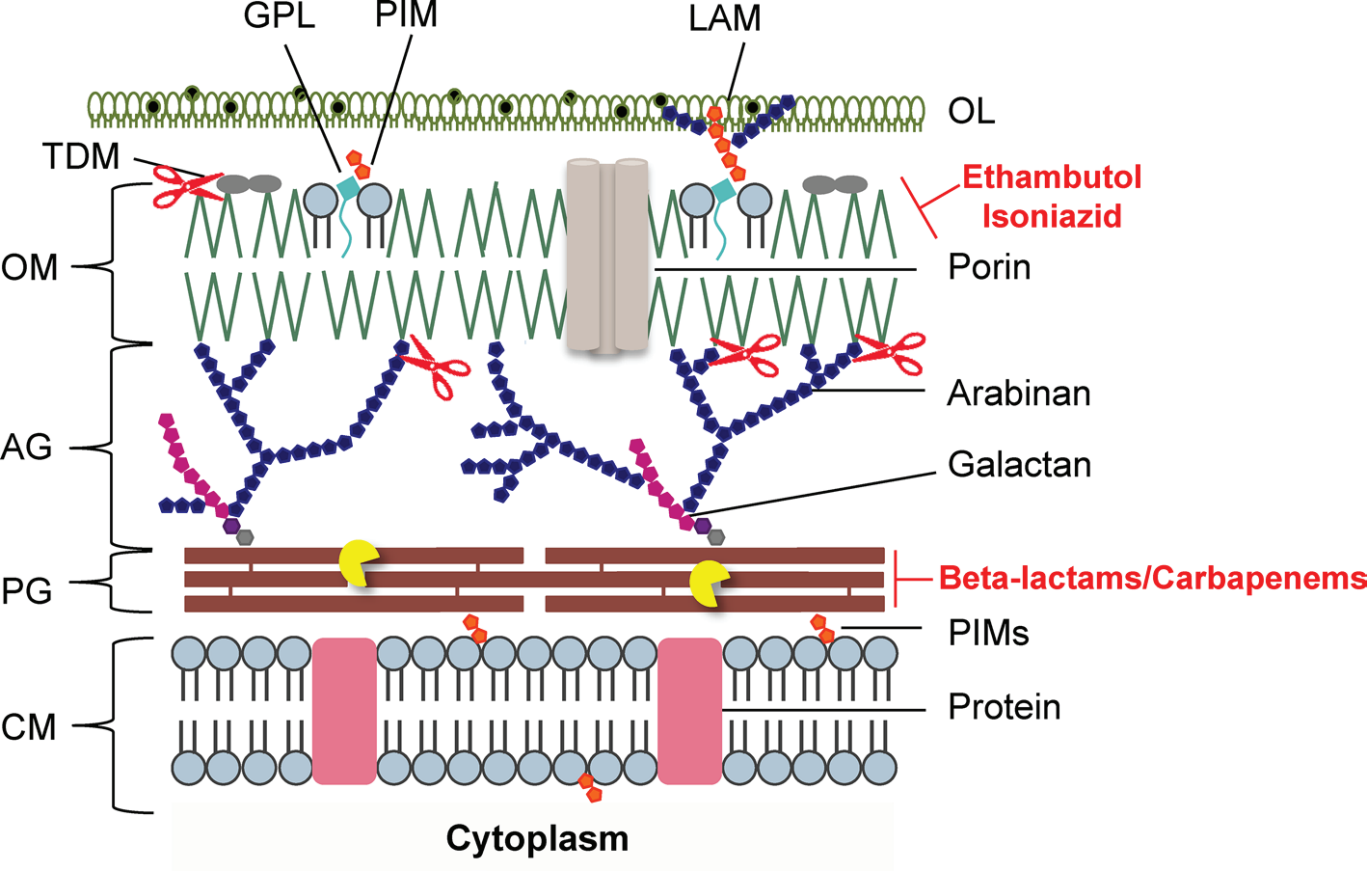
Schematic representation of the mycobacterial cell envelope layers. Inhibitors of mycolic acids and peptidoglycan biosynthesis are indicated in red. The mycobacteriophage lysis protein targets are indicated as follows: the pacman cartoon represents digestion of the PG by the endolysins; scissors illustrate LysB detachment of the OM. AG, arabinogalactan; CM, cytoplasmic membrane; GLP, glycolipids; LAM, lipoarabinomannan; OL, outer layer; OM, outer membrane; PG, peptidoglycan; PIMs, phosphatidylinositol mannosides; PLs, phospholipids; TDM, trehalose dimycolate. Adapted from Catalão and Pimentel, 2018 with permission from the authors.

PG biosynthesis (Figure 1) represents the site of action of the most widely used class of antibacterial agents for infection treatment (Vollmer et al., 2008; Bugg et al., 2011; Cho et al., 2014; Pavelka et al., 2014). However, except for d-cycloserine, an oral antimycobacterial agent that is specifically recommended by the WHO as a second-line anti-TB agent used as a last option for the treatment of TB (Hwang et al., 2013), antibiotics that target PG synthesis such as the β-lactams are only rarely used in the treatment of TB (Wong et al., 2013; Wivagg et al., 2014). This lack of efficacy against *Mtb* has primarily been attributed to a chromosomally encoded broad spectrum class A β-lactamase enzyme BlaC (Flores et al., 2005; Wang et al., 2006; Hugonnet and Blanchard, 2007), which hydrolyses the core β-lactam ring and deactivates the antibiotic, to different drug efflux pumps, to low affinity penicillin-binding proteins (PBPs) and to the expression of PG-biosynthetic enzymes insensitive to β-lactams (non-classical transpeptidases) (Wivagg et al., 2014; Gygli et al., 2017; Nasiri et al., 2017). In addition, the PG is camouflaged by the MA-rich mycobacterial OM that limits penetration of antibiotics (Figure 2; Brennan and Nikaido, 1995; Jankute et al., 2015).

### Resistance to d-Cycloserine in *Mtb*

d-cycloserine is a structural analog of d-alanine and interferes with the formation of PG biosynthesis, by acting as a competitive inhibitor of alanine racemase (Alr) and d-alanine-d-alanine ligase (Ddl) enzymes, which are involved in PG synthesis (Prosser and de Carvalho, 2013a,b,c). Ddl is the main target of d-cycloserine and is preferentially inhibited over Alr in *Mtb* (Prosser and de Carvalho, 2013a). Resistance to this antibiotic has been associated with loss-of-function mutations in metabolism-related genes of ubiquinone and menaquinone and *ald* (Rv2780), which encodes an l-alanine dehydrogenase (Hong et al., 2014; Desjardins et al., 2016). A recent study has identified novel mutations connected with d-cycloserine resistance in MDR and XDR *Mtb* strains, which demonstrate that resistance to this antibiotic is highly complex and involves diverse genes associated with different cellular processes such as lipid metabolism, methyltransferase, stress response, and transport systems (Chen et al., 2017). In another study, a genomic screening of more than 1,500 drug-resistant strains of *Mtb* revealed the presence of three main *alr* mutations (*alr_Mtb_* M319 T, *alr_Mtb_* Y364D, *alr_Mtb_* R373L) that confer d-cycloserine resistance (Nakatani et al., 2017). Despite the importance of d-cycloserine as a second-line drug used to treat MDR- and XDR-TB, the mechanisms underlying d-cycloserine resistance in *Mtb* clinical strains are still undetermined.

The emergence of MDR and XDR *Mtb* strains has become a serious health threat and has initiated the search for new therapeutic strategies. Some of those strategies include revisiting the potential use of β-lactams as an alternative therapeutic approach to tackle drug-resistant TB when no acceptable alternative exists (Hugonnet et al., 2009; Keener, 2014; Diacon et al., 2016).

### Resistance to β-Lactams in *Mtb*

Recent developments have led to the suggestion of using carbapenems, a modern class of extended-spectrum β-lactams, as the last line of defense against recalcitrant drug-resistant TB (Hugonnet et al., 2009; Payen et al., 2012; Gonzalo and Drobniewski, 2013; Davies Forsman et al., 2015; Jaganath et al., 2016; Payen et al., 2018). Among β-lactams, carbapenems are unique as they are not only relatively resistant to the hydrolytic activity of BlaC, but also act as potent inhibitors of this enzyme (Tremblay et al., 2010). The efficacy of carbapenems in killing *Mtb* is further increased by the ability of these compounds to inhibit the different enzymes that contribute to the assembly of mycobacterial PG (Gupta et al., 2010; Dubée et al., 2012; Erdemli et al., 2012; Cordillot et al., 2013; Bianchet et al., 2017; Kumar et al., 2017a). While most β-lactams inhibit d,d-transpeptidases (PBPs), which are the enzymes that catalyze the formation of 4 → 3 transpeptide linkages in the PG network (Zapun et al., 2008), they are unable to inhibit the l,d-transpeptidases (Ldts) that catalyze the formation of 3 → 3 transpeptide linkages. As the PG of mycobacteria contains a high proportion (up to 80%) of 3 → 3 cross-links (Lavollay et al., 2008; Cordillot et al., 2013), β-lactams will not fully prevent the assembly of the mycobacterial PG. Carbapenems inhibit not only d,d-transpeptidases but also l,d-transpeptidases (as well as d,d-carboxypeptidases) (Baranowski et al., 2018; García-Heredia et al., 2018).

Ldt and PBP enzymes are structurally unrelated and contain cysteine and serine residues in their active sites, respectively. *Mtb* genome encodes five l,d-transpeptidases, designated by Ldt_Mt1_ to Ldt_Mt5_ (Cordillot et al., 2013). It was shown that the presence of l,d-transpeptidases can markedly alter β-lactam susceptibility (Lavollay et al., 2008; Gupta et al., 2010; Dubée et al., 2012; Kumar et al., 2012; Cordillot et al., 2013; Kieser et al., 2015; Wivagg et al., 2016). In addition, recent studies indicate that *Mtb* strains that lack both *ldt_Mt1_* and *ldt_Mt2_* display enhanced susceptibility not only to amoxicillin, a β-lactam antibiotic, but also to vancomycin, a glycopeptide antibiotic (Schoonmaker et al., 2014). Furthermore, a synergistic effect of carbapenem with rifampicin was observed against rifampicin-resistant clinical isolates of *Mtb* (Kaushik et al., 2015, 2017).

Most of the anti-TB drugs associated with CW biosynthesis inhibition lack the ability to reduce treatment duration of TB drug regimens. This is related to the fact that some bacteria can withstand the presence of the antibiotics by becoming dormant, i.e., being unable to replicate, as dormant bacteria do not actively synthesize the CW and are presumably not affected by the presence of inhibitors of the CW synthesis. Recent research has shown that a combinatorial treatment that is based on the use of the β-lactamase inhibitor clavulanate and meropenem is effective against both actively replicating and non-replicating XDR *Mtb* isolates (Solapure et al., 2013). However, its high cost and intravenous administration present challenges to its widespread use. According to the WHO anti-tuberculosis classification, the carbapenems are included in Group D3, which indicates that safety and efficacy information to support its use against TB is restricted and should not be considered as an alternative regimen designated to treat TB (WHO, 2011, 2014). The existing *in vivo* and clinical studies suggest that there are advantages in carbapenem use as they are usually well-tolerated, although the variance in the extent of the treatment, dosing, and the absence of pharmacokinetic data limit interpretation of the effectiveness of these antibiotics against TB. Information regarding carbapenem resistance is scarce; mutations in CW biosynthesis genes and in *crfA* have been associated with resistance to different carbapenem antibiotics such as imipenem, meropenem, and biapenem (Lun et al., 2014; Kumar et al., 2017b). Nevertheless, these studies have been an enormous contribution to the recent and increased effort for repurposing β-lactams as an ultimate therapeutic option to treat life-threatening TB-infected patients and to unveil to what extent the wider *Mtb* human clinical isolates population may be susceptible to these antibiotics (Tiberi et al., 2016).

## Mycobacterial PG Assembly Enzymes as Targets for Antibiotics

The PG layer provides shape and rigidity to an individual cell of *Mtb* (Brennan, 2003). Since it is mainly restricted to bacterial cells, the enzymes that are involved in the biosynthesis of PG offer an attractive target for the development of new antibiotics against TB. In addition, the enzymes that catalyze the PG biosynthesis pathway in mycobacteria are essential, and therefore, their inhibition is expected to result in selective destruction of the bacteria (Moraes et al., 2015; Bhat et al., 2017; Abrahams and Besra, 2018). The biosynthesis process of mycobacterial PG is similar to other bacteria (Figure 1). The first step is catalyzed by the acetyltransferase and uridyltransferase activities of GlmU (Rv1018c), to yield UDP-Glc*N*Ac (Zhang et al., 2009). The functional resemblance of the GlmU uridyltransferase with human enzymes (Peneff et al., 2001) turns this enzyme into an unsuitable target (Rani and Khan, 2016). However, the lack of Glc*N*-1-P from mammals makes the acetyltransferase domain a promising target, and different substrate analogs of Glc*N*-1-P have been designed and shown to exhibit an inhibitory effect against GlmU by blocking synthesis of UDP-Glc*N*Ac (Figure 1; Li et al., 2011; Tran et al., 2013; Rani et al., 2015). The sequential MurA-F ligase pathway involves the formation of the UDP-*N*-acetylmuramic acid (UDP-Mur*N*Ac)-pentapeptide. MurA (Rv1315), a UDP-*N*-acetylglucosamine 1-carboxyvinyltransferase, and MurB (Rv0482), a UDP-*N*-acetylenolpyruvoylglucosamine reductase, are implicated in the formation of UDP-Mur*N*Ac. NamH (Rv3808), a UDP-*N*-acetylmuramic acid hydroxylase, hydroxylates UDP-Mur*N*Ac to UDP-*N*-glycolylmuramic acid (UDP-Mur*N*Glyc) in the cytoplasm to generate both types of UDP-muramyl substrates, although *Mtb* PG is enriched in the latter (Mahapatra et al., 2005; Raymond et al., 2005). Specific inhibitors of *Mtb* MurA and MurB have not been described to date. The broad-spectrum antibiotic, fosfomycin, which targets Gram-negative MurA, has no activity against *Mtb* since the critical cysteine (Cys_117_) residue, which is required for inhibition by the drug, is replaced in *Mtb* by an aspartic acid residue, contributing to the intrinsic resistance against this antibiotic (Kim et al., 1996). A limited number of inhibitors have been reported against MurB, specifically the 3,5-dioxopyrazolidine and benzylidene thiazolidinedione derivatives which can competitively inhibit the formation of UDP-Mur*N*Ac (Figure 1; Kumar et al., 2011; Rana et al., 2014). Inhibitors of NamH have not been reported, probably due to the fact that *namH* is not essential in *Mtb* (Hansen et al., 2014). Therefore, NamH may not be a key target for anti-TB therapy. However, *Mtb* strains that lack *namH* are hypersusceptible to β-lactam antibiotics, and therefore, inhibitors of NamH could potentiate the effect of carbapenems (Raymond et al., 2005; Hansen et al., 2014). From this point, the pentapeptide chain is attached to UDP-Mur*N*Ac/Glyc by the ATP-dependent Mur ligases (Figure 1), beginning with UDP-*N*-acetylmuramoyl-l-alanine addition by MurC (Rv2151c). This is followed by d-isoglutamate addition by MurD (Rv2155c), m-DAP addition by MurE (Rv2158c), and finally d-alanyl-d-alanine addition by MurF (Rv2157c). This generates the muramyl-pentapeptide product UDP-Mur*N*Ac/Glyc-l-Ala-d-isoGlu-*m*-DAP-d-Ala-d-Ala, also known as Park’s nucleotide (Figure 1; Pavelka et al., 2014). Several inhibitors of the Mur ligases have been identified (Hrast et al., 2014). One example is the substituted 5-benzylidenethiazolidin-4-one derivatives that inhibit the formation of the pentapetide chains (Tomasic et al., 2010). However, their utilization is limited against *Mtb* Mur ligases given that only MurC and MurE have been biochemically characterized (Mahapatra et al., 2000; Li et al., 2011). Ddl is the target of d-cycloserine, a structural analog of d-Ala, inhibiting the binding of either the two d-Ala substrates to Ddl (Bruning et al., 2011; Prosser and de Carvalho, 2013c). The assembled Park’s nucleotide is then transferred to undecaprenyl phosphate present at the membrane by MraY (Rv2156c) generating Lipid I. Nucleoside-peptide antibiotics that inhibit MraY have been described, including muramycin, liposidomycin, caprazamycin, and capuramycin (Dini, 2005; Wiegmann et al., 2016; Tran et al., 2017). Remarkably, capuramycin has been shown to kill non-replicating *Mtb*, an uncommon characteristic of the majority of CW biosynthesis inhibitors (Koga et al., 2004; Reddy et al., 2008; Nikonenko et al., 2009; Siricilla et al., 2015). The final intracellular step of PG synthesis is catalyzed by MurG, a glycosyltransferase that is responsible for producing lipid II, the final monomeric block of PG. An *Escherichia coli* designed inhibitor of MurG was tested against *Mtb* with limited success and has become the first inhibitor identified against the *Mtb* glycosyltransferase (Trunkfield et al., 2010).

Translocation of lipid II across the plasma membrane is carried out by a flippase. This was initially thought to be an FtsW-like protein, Rv2154c (Mohammadi et al., 2011). However, recent research has shown that FtsW/RodA enzymes elongate PG chains through a transglycosylase activity (Meeske et al., 2016; Emami et al., 2017), and therefore, the best candidate for the PG precursor flippase is currently MurJ (Rv3910). Following the transport of PG precursor across the mycobacterial membrane, the bifunctional PonA1/PBP1 (Rv0050) and PonA2/PBP2 (Rv3682) enzymes, PBPs that possess both the transglycosylase and transpeptidase domains attach the Glc*N*Ac moiety to the muramyl moiety of the nascent PG chain (Figure 1). Lipid II inhibitors, such as the depsipeptide antibiotics ramoplanin and enduracidin (Fang et al., 2006) and teixobactin (Ling et al., 2015), that prevent the transglycosylation of the translocated lipid II by binding to it have been described recently. The transpeptidase activity of PonA1 and PonA2 catalyzes the classical 4 → 3 cross-linkages between m-DAP and d-Ala of the adjacent pentapeptide chains present in neighboring glycan chains, with the cleavage of the terminal d-Ala. Other d,d-transpeptidation and d,d-carboxypeptidation reactions are catalyzed by the monofunctional PBPs, both resulting in the cleavage of the terminal d-Ala of the peptide stem (Zapun et al., 2008). Among the muropeptides present in the *Mtb* PG, up to 80% of the cross-links are 3 → 3 links between m-DAP residues of two adjacent tetrapeptide stems, with the release of the fourth position d-Ala (Lavollay et al., 2008), performed by non-classical l,d-transpeptidases, Ldt_Mt1_ (Rv0116c), Ldt_Mt2_ (Rv2518c), Ldt_Mt3_ (Rv1433), Ldt_Mt4_ (Rv0192), and Ldt_Mt5_ (Rv0483) (Lavollay et al., 2008; Cordillot et al., 2013). As mentioned before, the l,d-transpeptidase and d,d-carboxypeptidase activities are unaffected by most β-lactam antibiotics, except the carbapenems (Gupta et al., 2010; Dubée et al., 2012; Kumar et al., 2012; Cordillot et al., 2013; Rullas et al., 2015; Bianchet et al., 2017; Kumar et al., 2017a). Moenomycin, a glycolipid that inhibits the transglycosylase activity of PBPs (van Heijenoort et al., 1987) is yet to have recognized efficacy against *Mtb*. The existence of other antibiotics that act on the availability of PG precursors: (1) the glycopeptides, vancomycin and teicoplanin, that bind to the d-Ala-d-Ala terminus of the pentapeptide stem and prevent PG polymerization (Reynolds, 1989); (2) the lantibiotic family of antibiotics, such as nisin, that interact with the pyrophosphate moiety of lipid II, with the consequent delocalization of this molecule that can form a pore in the cytoplasmic membrane and inhibit PG biosynthesis (Wiedemann et al., 2001), opens new avenues to find suitable synergistic antibiotic combination schemes for effective treatments.

## Prospective use of Mycobacteriophage Endolysins to Degrade the Mycobacterial PG

The mycobacterial PG is modified by several enzymes, which confer resistance to some widely used antibiotics (Mahapatra et al., 2005; Raymond et al., 2005). Mycobacteriophages, the viruses of mycobacteria, synthesize enzymes to eliminate each layer of the cell envelope (recently reviewed in Catalão and Pimentel, 2018), so that phage particles can escape from the bacterial cell at the end of a replicating cycle. Mycobacteriophage-encoded PG hydrolases (endolysins) are predicted to target and degrade nearly every bond in mycobacterial PG (Figure 1; Payne and Hatfull, 2012; Catalão et al., 2013; Pimentel, 2014). Given the essentiality of the mycobacterial cell envelope (Brennan and Nikaido, 1995; Jankute et al., 2015; Chiaradia et al., 2017), it is reasonable to consider that the enzymatic degradation of mycobacterial CW by the mycobacteriophage lytic enzymes (Gil et al., 2008, 2010; Payne et al., 2009; Catalão et al., 2011; Gigante et al., 2017) may be a promising therapeutic approach to kill extracellular pathogenic mycobacteria (Grover et al., 2014) or after their internalization by macrophages (Lai et al., 2015). However, access of mycobacteriophage endolysins to their substrate, the PG, is hindered by the MA-rich mycobacterial OM, which restrains their use as anti-TB therapeutic agents (Figure 2). Therefore, transport of phage enzymes and/or antibiotics that target the PG metabolism through this OM remains the major constraint in the application of these compounds in therapy (Catalão and Pimentel, 2018). As the enzymes involved in MA and AG biosynthesis and integrity play an important role in the development of drug resistance in *Mtb*, inhibition of the synthesis of these CW layers would damage the CW as a barrier, increase its permeability, and increase the susceptibility of bacteria to various anti-mycobacterial drugs (Figure 2). Mycobacteriophage-encoded LysB proteins are specific lysis proteins that act enzymatically, not only hydrolyzing lipids on the outer leaflet of the OM, but also, importantly, detaching it from the CW by cleaving the ester linkage to the AG polymer due to a mycolyl-arabinogalactan esterase activity (Figure 2; Gil et al., 2008, 2010; Payne et al., 2009; Gigante et al., 2017). Interestingly, it has been recently reported that ethambutol, one of the first-line drugs for TB treatment, leads to the loss of the MA layer by blocking polymerization of arabinose in AG, which impairs *de novo* synthesis of the outer envelope layers (Schubert et al., 2017). As cell division seems to be unaffected by ethambutol, the authors proposed that the inhibition of MA synthesis generates a defective CW composed predominately of exposed PG. Inactivation of the Ag85 complex (*fbpA*, *fbpB*, and *fbpC*) proteins that possess mycolyltransferase activity and are involved in biogenesis of trehalose dimycolate (TDM), a glycolipid that has been proposed to be present in the outer leaflet of the mycobacterial OM, increased sensitivity both to first-line TB drugs and to erythromycin, imipenem, rifampicin, and vancomycin (Lingaraju et al., 2016). Since production of TDM by Ag85 is essential for the intrinsic antibiotic resistance of mycobacteria (Morris et al., 2005), Ag85-specific inhibitors or TDM hydrolysis by LysB (Figure 2; Gil et al., 2010) can have a positive impact on the fight to control mycobacterial drug resistance.

Understanding the mechanisms used by mycobacteriophages to deteriorate each layer of the extremely complex mycobacterial cell envelope is highly relevant for the design of new strategies against mycobacteria. Given the abundance of isolated mycobacteriophages, which constitute an enormous reservoir of CW degrading enzymes capable of hydrolyzing each specific linkage of the mycobacterial cell envelope (Hatfull, 2006; Payne and Hatfull, 2012), it is worth to consider the possibility of using these enzymes in synergistic combinations with CW targeting antibiotics, which have a limited access to their target, the PG in normally growing bacteria (Figure 2).

## Concluding Remarks

Inhibition of the assembly of the bacterial CW by anti-mycobacterial agents that successfully target the synthesis of its various components has proven useful in tackling TB (Wong et al., 2013; Bhat et al., 2017). However, modification of CW targets mediated by specific enzymes or the accumulation of chromosomal mutations and degradation/modification of drugs by production of antibiotic inactivating enzymes has rendered *Mtb* resistant to most classes of antimicrobials (Eldholm and Balloux, 2016; Gygli et al., 2017; Nasiri et al., 2017). Infections due to *Mtb* are an increasing problem worldwide, and the emergence of XDR-TB suggests that *Mtb* may become refractory to any chemotherapeutic agent in the future (Horsburgh et al., 2015; World Health Organization, 2017). The limited number of new anti-mycobacterial agents approved for therapy and the wide variety of *Mtb* intrinsic and acquired drug resistance mechanisms to the available drugs have contributed to an increased effort to repurpose the use of antibiotics that are not commonly used in anti-TB therapy and to find suitable synergistic antibiotic combinations for effective treatment of life-risk TB (Mainardi et al., 2011; Wong et al., 2013; Keener, 2014; Diacon et al., 2016). Recent studies have uncovered the possibility of targeting the mycobacterial PG biosynthesis and degradation as an alternative option for anti-TB therapy (Tomasic et al., 2010; Trunkfield et al., 2010; Li et al., 2011; Rana et al., 2014; Ling et al., 2015; Rani et al., 2015; Rullas et al., 2015; Tran et al., 2017). In addition, several observations suggest that inhibition of PG synthesis by transpeptidase inhibitors such as the carbapenems or glycopeptide antibiotics could synergize with other CW inhibitors and increase their efficacy (Figures 1 and 2; Hugonnet et al., 2009; Kumar et al., 2012; Kieser et al., 2015; Schubert et al., 2017). The recent developments toward the potential application of mycobacteriophage-dedicated enzymes targeting the complex mycobacterial CW arrangement have also renewed the interest of repurposing mycobacterial PG metabolism as an anti-TB therapy target (Gil et al., 2008, 2010; Payne and Hatfull, 2012; Catalão and Pimentel, 2018). More research is needed in the near future that could lead to the design and development of therapeutics that increase the efficacy of currently available antibiotics and enzymes that target PG metabolism, which is not currently considered as an alternative to treat TB.

## Author Contributions

MC and MP conceived and designed the study and wrote the manuscript. MC, SF, and MP participated in manuscript revising and editing.

### Conflict of Interest Statement

The authors declare that the research was conducted in the absence of any commercial or financial relationships that could be construed as a potential conflict of interest.
